# Thermal evolution of gene expression profiles in *Drosophila subobscura*

**DOI:** 10.1186/1471-2148-7-42

**Published:** 2007-03-19

**Authors:** Hafid Laayouni, Francisco García-Franco, Blanca E Chávez-Sandoval, Vincenzo Trotta, Sergi Beltran, Montserrat Corominas, Mauro Santos

**Affiliations:** 1 Departament de Genètica i de Microbiologia, Grup de Biologia Evolutiva (GBE), Universitat Autònoma de Barcelona, 08193 Bellaterra (Barcelona), Spain; 2 Unitat de Biologia Evolutiva, Departament de Ciències de la Salut i de la Vida, Universitat Pompeu Fabra, Doctor Aiguader 80, 08003 Barcelona, Spain; 3 Dipartimento di Biologia Evoluzionistica Sperimentale, Università di Bologna, Via Selmi 3, 40126 Bologna, Italy; 4 Departament de Genètica, Facultat de Biologia, Universitat de Barcelona, Av. Diagonal 645, edifice annex, 1^a ^planta, 08028 Barcelona, Spain; 5 Centre de Regulació Genòmica (CRG), Doctor Aiguader 88, 08003 Barcelona, Spain

## Abstract

**Background:**

Despite its pervasiveness, the genetic basis of adaptation resulting in variation directly or indirectly related to temperature (climatic) gradients is poorly understood. By using 3-fold replicated laboratory thermal stocks covering much of the physiologically tolerable temperature range for the temperate (i.e., cold tolerant) species *Drosophila subobscura *we have assessed whole-genome transcriptional responses after three years of thermal adaptation, when the populations had already diverged for inversion frequencies, pre-adult life history components, and morphological traits. Total mRNA from each population was compared to a reference pool mRNA in a standard, highly replicated two-colour competitive hybridization experiment using cDNA microarrays.

**Results:**

A total of 306 (6.6%) cDNA clones were identified as 'differentially expressed' (following a false discovery rate correction) after contrasting the two furthest apart thermal selection regimes (i.e., 13°C *vs *. 22°C), also including four previously reported candidate genes for thermotolerance in *Drosophila *(*Hsp26*, *Hsp68*, *Fst*, and *Treh*). On the other hand, correlated patterns of gene expression were similar in cold- and warm-adapted populations. Analysis of functional categories defined by the Gene Ontology project point to an overrepresentation of genes involved in carbohydrate metabolism, nucleic acids metabolism and regulation of transcription among other categories. Although the location of differently expressed genes was approximately at random with respect to chromosomes, a physical mapping of 88 probes to the polytene chromosomes of *D. subobscura *has shown that a larger than expected number mapped inside inverted chromosomal segments.

**Conclusion:**

Our data suggest that a sizeable number of genes appear to be involved in thermal adaptation in *Drosophila*, with a substantial fraction implicated in metabolism. This apparently illustrates the formidable challenge to understanding the adaptive evolution of complex trait variation. Furthermore, some clustering of genes within inverted chromosomal sections was detected. Disentangling the effects of inversions will be obviously required in any future approach if we want to identify the relevant candidate genes.

## Background

Temperature is a fundamental feature that affects all living organisms. Each species, particularly ectotherms, has a non-stressful thermal tolerance range and responds to temperature by physiological, biochemical, and molecular level adjustments that underlie adaptation. For instance, many latitudinal clines exist in *Drosophila *for allele frequencies at allozyme loci, chromosomal inversions, and microsatellites; as well as for traits like starvation resistance, desiccation resistance, and body size where the differences between populations have a genetic basis and can even persist for many generations under laboratory reared conditions [1-4]. By and large, the empirical evidence suggests that variation in these markers and traits are directly or indirectly related to temperature (climatic) gradients. Perhaps the most pertinent example comes from recent studies on chromosomal inversion polymorphisms showing that the genetic constitution of populations is responding to contemporary rapid global warming [5-8].

The native Palearctic fly *Drosophila subobscura *spans more than 30° latitude in the Old World: from North Africa to Scandinavia. As a result, its populations experience a strong climatic gradient [9]. In the late 1970s and early 1980s the species invasively spread in North and South America and nowadays spans about 15° latitude on each continent [10,11]. Remarkably enough, latitudinal clines in the New World for inversion polymorphism and body size parallel to the long standing ones in the native geographic area were evident after a few years since the American colonization. This 'replicated time series experiment of evolution in action' [12,13] strongly suggests that those traits are indeed subject to selection from temperature-related factors. However, a laboratory natural selection experiment (i.e., an experimental protocol where stocks of organisms are reared under different conditions and allowed to evolve by natural selection over many generations [14]) specifically designed to test the putative role of temperature *per se *in the evolution of these clines found results conflicting to those expected from the latitudinal gradients for both inversions and body size [3,15,16]. Certainly, laboratory experiments are not the best way to mirror what is happening at different latitudes and to reconstruct natural clines. But at present it is unclear whether temperature alone drives the clines. What types of genetic changes are needed for an organism to adapt to new thermal conditions?

A number of authors (e.g. [17,18]) have argued that changes in the transcriptome constitute a major component of the genetic basis for phenotypic evolution. Gene expression profiling by means of microarrays has become a popular way of finding candidate genes of trait variation and is providing new insights into some old but fundamental questions in evolutionary biology [19-22]. Here we examine global gene expression by measuring the relative abundance of mRNAs in third instar larvae of *D. subobscura *from 3-fold replicated laboratory thermal selection stocks -derived from the estimated Chilean epicentre (Puerto Montt) of the original New World invasion [15]- that had evolved at three constant temperature regimes during 3 years: cold (13°C), optimum (18°C) and warm (22°C). The connection between the very high dimensional nature of the gene-expression data and the multivariate whole organism phenotype, however, is not straightforward and detailed functional and ecological analyses of candidate genes will obviously be required to understand the genetic basis for thermal adaptation (e.g., [23]).

In holometabolous insects like *Drosophila *many adaptations to changing environments involve changes in larval behaviour and physiology that may impinge on other phases of the life cycle (e.g. [24-26]). For this reason we used third instar larvae for the microarray experiment. A possible drawback was that both sexes were mixed, so we have overlooked any sex-specific thermal response that might have been present. Total mRNA from each population was compared to a reference pool mRNA independently derived from the optimum (P18) populations (see Methods) in a standard two-colour competitive hybridization using cDNA microarrays with *D. melanogaster *clones [27]. Heterologous hybridization to study gene expression profiles has been validated between closely related species (divergence time < 10 Mya), and consistent data are also obtained for less closely related taxa (divergence time ~65 Mya; [28]). *Drosophila subobscura *belongs to the *D. obscura *group, and the divergence time between the *D. melanogaster *and *D. obscura *groups has been estimated to be ~25 Mya [29]. This apparently offers a reasonable warranty to use *D. melanogaster *arrays in heterologous hybridization with *D. subobscura*. In addition, we carried out some preliminary tests in order to optimize the experimental conditions. Highly reproducible and consistent gene profiling, comparable to that obtained with homologous hybridization by using some *D. subobscura *clones added to the arrays (see Methods), was observed.

It is also important to remark here that the lower and upper thermal regimes used in the experiment are not stressful: the temperature range likely covers much of the physiologically tolerable range in this species [9]. Obviously, the constant thermal regimes and light:dark period where the populations have evolved (see Methods) do not mirror the seasonal changes experience by natural populations, but with this experimental protocol we can control that temperature is the only factor differing between the thermal stocks. The thermal stocks had already diverged for inversion frequencies, pre-adult life history components, and morphological traits [3,16]. The experimental design equated to a four-way factorial analysis of variance (ANOVA) with thermal selection regime and cyanine dyes (Cy3, Cy5) in a flip dye design as fixed effects, replicated populations as a random factor nested in thermal selection regime, and slide (spotted microarray) as a random factor nested in thermal selection, dye, and replicate. The analysis allowed identifying quantitative differences in larval gene expression between cold- (P13) and warm-adapted (P22) populations.

The candidate genes were assigned a biological function and/or biological process when information was available. Also important, a number of genes were mapped by *in situ *hybridization to the polytene chromosomes of *D. subobscura*. The karyotype of this species consists of five acrocentric chromosomes and a dot chromosome (see Methods). What is crucial here is that *D. subobscura *harbours one of the richest inversion polymorphisms in the genus, with a total of 92 chromosomal arrangements (produced from 66 inversions located on all major chromosomes) recorded in the native Palearctic region [9,30]. This number reduces to 18 arrangements in colonizing populations of the New World, all of them but one segregating in the thermal stocks [16]. Variation in some traits is known to be tied to inversion polymorphisms in *Drosophila *(e.g. [31,32]), and quantitative associations between larval gene expression and thermal adaptation could be due to position effects (e.g., the inversion of a chromosomal segment can remove or exchange the regulatory sequences of a gene and alter its expression pattern [33]) or hitchhiking arisen from linkage disequilibrium. In view of the rapidly and consistently evolved latitudinal clines in chromosome inversion polymorphism following the New World invasion by the species [12], and the shifts in inversion frequencies in response to laboratory thermal adaptation [16] and to climate change [7], we expect that a large number of genes will be included inside inverted chromosome segments. Linkage with inversions will highly complicate the identification of chromosome regions that are targets of selection.

## Results and discussion

### Overall patterns of gene expression in the thermal lines

An important point in the experiment was that the parents of treatment larvae had also been reared at the same temperature of 18°C to control for the possibility of non-genetic parental effects on offspring (see Methods). In order to generate a reliable data set we analyzed mRNA abundance from a highly replicated experiment: four independent batches of 250 optimum-reared larvae each -amounting to 9,000 larvae in total (i.e., 250 larvae × 4 slides per population × 9 experimental populations)- whose mRNAs were competitively hybridized to a reference pooled mRNA from 9,000 control larvae on contiguous duplicated gene spots using a dye-reversal experimental design, thus providing up to 72 gene expression values for each probe. Furthermore, some genes were spotted (in duplicated) several times on the slides, which helped to confirm the quality and consistency of the data as there was a clear correspondence among different spots [see Additional file 1: summary of the microarray results].

**Figure 1:**
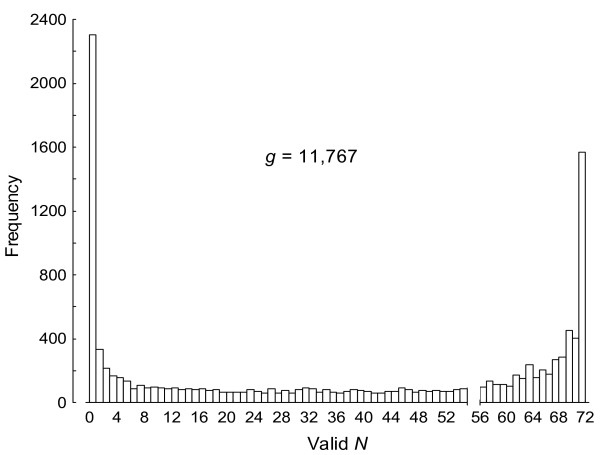
**Distribution of valid gene expression levels**. For a given probe *g *the maximum number of valid gene expression values for each thermal selection regime was *N *= 72. All probes with *N *< 57 (left part just before the *x*-axis scale break) were excluded from the statistical analysis.

**Figure 2:**
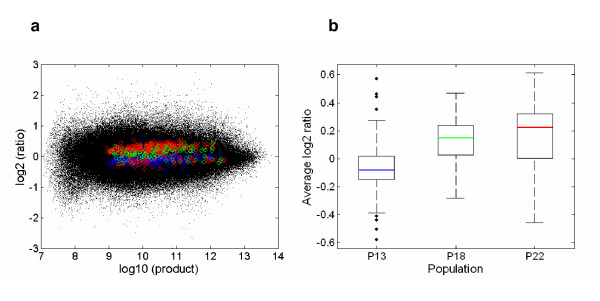
**Microarray signature**. Gene expression profiles in the three thermal regimes. **(a) **Expression ratio versus fluorescence intensity of the 316,229 spots (black dots) from the 4,651 clones approved for statistical analyses. The averages for those probes identified as differentially expressed when contrasting the two extreme thermal regimes (i.e.,13°C *vs*. 22°C) are in blue (P13), green (P18) and red (P22). **(b) **Box-plots of the average expression ratios for the differentially expressed genes.

The distribution of normalized measures for valid relative gene expression levels in the *g *= 11,767 cDNA clones is shown in Fig. 1. All clones with less than 57 valid expression observations were excluded from further analyses and, therefore, we will only focus on the right part of Fig. 1 just after the *x*-axis scale break. As a result, 4,651 cDNA clones (4,310 non-redundant genes) were allowed for differential gene expression analysis and their microarray signature, plotted as expression ratio versus fluorescence intensity, is shown in Fig. 2a. For each clone *g *= 1, ⋯, 4,651 the dye-reversal experimental design was subjected to a least squares ANOVA model as that shown in Table 1 for (e.g.) gene *CG12236*. A key premise in the experimental design with 2 degrees of freedom for the main fixed factor temperature was to define *a priori *the linear contrast between the two furthest apart thermal selection regimes: warm (22°C) *vs*. cold-adapted (13°C) populations (each comparison or contrast between two means has one degree of freedom).

**Table 1:** ANOVA of relative intensity ratios for gene *CG12236*.

Source of variation	d.f.	Sum of Squares	Mean Square	*F*	*p*-value (parametric)	*p*-value (permutation)
Temperature (T)	2	1.3882	0.6941	11.475	0.009	0.008
P22 *vs*. P13	1	1.2829	1.2829	21.210	0.004	0.002
Replicate^a^	6	0.3629	0.0605	0.346	0.905	
Dye (D)	1	3.5337	3.5337	20.193	< 0.001	
T × D	2	0.0392	0.0196	0.112	0.895	
Slide^b^	24	4.1998	0.1750	11.816	< 0.001	
Error^c^	36	0.5332	0.0148			

Temperature stands for the fixed effects due to thermal selection regime (13, 18 and 22°C), replicate for the random effect of replicated populations (R1, R2 and R3) nested in thermal regime, dye for the fixed effect of cyanine dye Cy3 (green) or Cy5 (red) in the flip dye design, and slide for the random effect of cDNA microarray glass slide nested in thermal regime, replicate and dye.

^a ^Error term for temperature and for the planned contrast comparing warm- (P22) *vs*. cold-adapted (P13) populations. ^b ^Error term for replicate, dye, and temperature × dye interaction. ^c ^Error term for slide.

A total of 419 (9%) cDNA clones were identified as 'differentially expressed' when considering a *p*-value (from 10,000 rounds of permutation) cut-off of 5% for the temperature factor with 2 degrees of freedom (see Table 1), but none of them was labelled as truly significant in terms of the false discovery rate (FDR; [34]) method used in detecting differential gene expression (*q*-value threshold of 5%; see Methods). On the other hand, from the permutation *p*-values obtained after the linear contrasts between the two furthest apart thermal selection regimes the number of 'differentially expressed genes' rose up to 950 (20.4%), with 306 (6.6%) remaining significant after a FDR correction (recall that a *q*-value threshold of 5% means that among all genes considered as significant, 5% of these are truly null on average [35]). Fig. 2 also shows the averages for the expression ratio versus fluorescence intensity of the identified 306 genes differing in gene expression (Fig. 2a), together with the corresponding box-plots (Fig. 2b). The reason why the linear contrasts comparing P22 *vs*. P13 populations yielded more differentially expressed genes was because the averages of log 2 relative intensity ratios for optimum (P18) populations were normally in between the averages for P13 and P22 populations. Therefore, a substantial proportion of the sum of squares for the temperature factor with 2 degrees of freedom in the ANOVAs was accounted for by the linear contrast (e.g., ~92% in Table 1).

**Figure 3:**
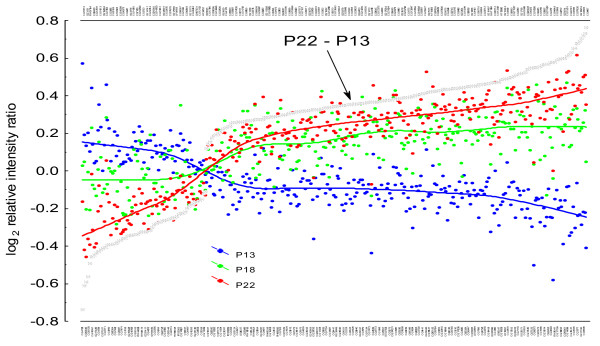
**Transcriptome changes following thermal adaptation**. Scatterplots of the average log2 relative intensity ratio for the 306 'differentially expressed genes' detected from the linear contrasts between the two furthest apart thermal selection regimes (i.e., 13°C *vs*. 22°C) and sorted according to the difference between warm- and cold-adapted (i.e., P22 – P13) populations. The sorted CG names of genes are given at the bottom (1, 3, 5, ⋯) and top (2, 4, 6, ⋯) axes. For each gene, the points in blue (P13), green (P18) and red (P22) give the average log2 relative intensity ratio for the different thermal regimes. The corresponding points have been connected by polynomial fitting to enhance visibility.

A far more informative plot is shown in Fig. 3, where gene expression values are sorted according to the contrast estimates. It is clear that those populations that have evolved at the optimum thermal regime (18°C) ranged in between, and that the average gene expression difference between warm- and cold-adapted populations was generally low: from 0.6 for *CG4183 *(heat shock protein 26; *Hsp26*) to 1.7 for *CG4867 *(*bc10*).

### Gene expression grouped by cellular function and biological process

**Figure 4:**
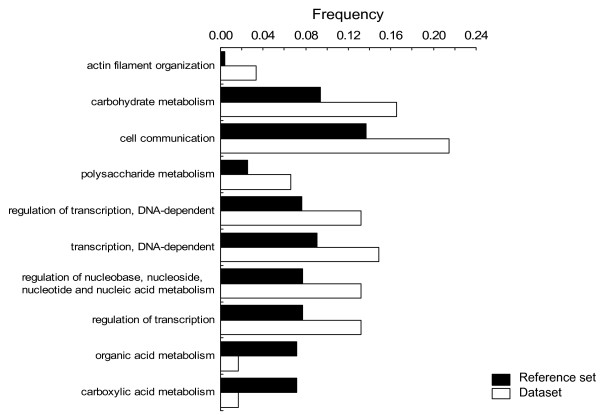
**Genes grouped by gene ontology (GO) terms**. For all statistical significance categories, the bars in the reference set plot the subset frequencies of annotated genes in the cDNA microarrays belonging to a particular class with valid gene expression data for statistical analysis (Fig. 1), and the bars in the dataset represent those frequencies in the group of genes labeled as 'differentially expressed' when contrasting the two extreme thermal regimes. Two categories are apparently underrepresented (organic acid and carboxylic acid metabolism), whereas the rest are overrepresented in the dataset [see Additional file 2].

Analysis of functional categories defined by the Gene Ontology project [36] using the GOToolBox [37] revealed that our reference dataset (the 4,651 cDNA clones with 4,310 non-redundant genes that were allowed for differential gene expression analysis) includes 989 annotated genes, and only 66 out of 306 genes labelled as 'differentially expressed' were annotated. These genes could be assigned to different cellular or molecular functions: over two-thirds are involved in metabolism processes (41 genes), in transport processes (14 genes), and in regulation of transcription (8 genes). (Note that many genes belong to more than one category.) For each functional category, we compared the actual number of occurrences with the expected one under the null hypothesis that all categories should be equally represented. Namely, the probability of obtaining by chance a number *n *of annotated genes for a given term among a dataset of size *N*, knowing that the reference dataset contains *m *such annotated genes out of *G *genes, is calculated. This test follows the hypergeometric distribution and the GOToolBox allows for FDR correction, pointing at statistically relevant over- or underrepresented terms within a dataset. The results obtained are shown in Fig. 4 and indicate an overrepresentation of genes involved in carbohydrate metabolism, nucleic acids metabolism and regulation of transcription among other categories. Two categories are apparently underrepresented: organic acid and carboxylic acid metabolism [see Additional file 2: molecular function gene ontology categories of differentially expressed genes].

It seems reasonable that genetic adjustments to environmental differences may involve changes in metabolism. Thus, when inbred and non-inbred *D. melanogaster *lines are reared under benign and stressful (high temperature) environmental conditions gene expression patterns of metabolic genes are strongly affected by both inbreeding and temperature stress [38]. Furthermore, previous studies on thermal evolution using the same *Drosophila *species have shown that differences between cold- and warm-adapted populations can be due to differences in the efficiency of larval growth [24,39]. In our thermal stocks with *D. subobscura *we have shown that cold-adapted (P13) populations had longer development times in the whole range of developmental temperatures assayed, and that warm-adapted (P22) populations seem to have evolved faster development [3]. Together with the lack of divergence for adult body size, it seems that cold-adapted *D. subobscura *stocks achieve the 'target' size by growing more slowly. This apparently agrees with their lower level of gene expression for genes involved in metabolic processes when compared to their warm-adapted counterparts.

### Candidate genes for thermotolerance in Drosophila

Expression levels for genes of the heat shock protein group (Hsps) that act as molecular chaperones and are important for cellular housekeeping are known to covary with the thermal regimes experience by populations, species and higher taxa [40]. Hsp70 appears to be the primary protein involved in thermotolerance in *D. melanogaster *[41] -though apparently not in other *Drosophila *species [42]-, and *Hsp70 *allele frequencies show latitudinal clines and change in response to thermal evolution in the laboratory [43]. In addition, *Hsp23 and Hsp26 *latitudinal variation in the *D. melanogaster *Australian cline [44], and correlated responses to selection for knockdown resistance at 39°C for *Hsp68 *[45], have also been found. Besides Hsps, other candidate genes for adaptation to thermal extremes (summarized in [46]) are: *Hsrω *(heat-shock RNA *ω*, which produces two RNA products but no known protein product [47]), *Hsf *(heat-shock transcription factor), *Tot *(*Turandot*), *mth *(*methuselah*, also a candidate aging gene [48,49]), *Dca *(*Drosophila *cold acclimation gene), *Fst *(*frost*; involved in recovering from cold shock [50]), *Drs *(*drosomycin*), *shark *(involved in a signaling pathway for epithelial cell polarity [51]), *anon-23Da *(encoding a protein with currently unknown function), *desat2 *(*desaturase2*; [52]), *period *(clock gene that determines biological rhythmicity in *Drosophila *[53]), *Ddc *(dopa decarboxylase; involved in the catecholamine biosynthesis pathway, which has been implicated in the response to various stressors including temperature [54]), and various metabolic enzymes as *Adh *(alcoholdehydrogenase; e.g., [55,56]), *Gpdh *(Glycerol 3 phosphate dehydrogenase; [56]), *Gdh *(NAD-dependent glutamate dehydrogenase; [57]) and *Treh *(trehalase; [57]).

We had valid gene expression values (i.e., *N *≥ 57 in Fig. 1) for all but 6 of the formerly listed genes; namely, *Hsp70*, *Hsp23*, *Hsp26*, *Hsp68*, *Hsf*, *Fst*, *shark*, *anon-23Da*, *period*, *Ddc*, *Gpdh *(*D. subobscura *clone added to the microarrays), *Gdh *and *Treh*. (We also had valid expression data for *desat1 *but not for *desat2*.) Differential gene expression with the *q*-value cut-off chosen for the linear contrasts between cold- (P13) and warm-adapted (P22) populations was found only for *Hsp26*, which showed increased expression levels in P13 populations. However, the *q*-value thresholds for *Hsp68 *(0.058; P13 > P22), *Fst *(0.061; P13 < P22), and *Treh *(0.061; P13 < P22) were low enough as to suggest that these three candidate genes also diverged in gene expression levels in our populations. (*q*-value thresholds for the other genes were: *Hsp70*, 0.506; *Hsf*, 0.505; *shark*, 0.293; *anon-23Da*, 0.135; *desat1*, 0.148; *period*, 0.505; *Dcd*, 0.353; *Gdh*, 0.096 [see Additional file 1].) It seems, therefore, that our survey in *D. subobscura *apparently links thermal adaptation in a temporally stable environment to some specific candidate genes (*Hsp26*, *Hsp68*, *Fst*, and *Treh*) previously associated to thermotolerance in *D. melanogaster*. It is also worth saying that the *Hsp60 *gene had a *q*-value threshold of 0.082 (P13 > P22) and might have also been associated with thermal adaptation. On the other hand, *Hsp83 *is known to be expressed during normal development in *D. subobscura *but increased transcription occurs when flies are reared at heat-shock temperatures from 26 to 34°C [58]. Consistent with this finding we did not observe a statistically significant differential gene expression between cold- and warm-adapted populations for *Hsp83 *(*q*-value threshold 0.467).

### Mapping of differentially expressed genes by in situ hybridization and correlated expression patterns

*In situ *hybridizations to the polytene chromosomes were routinely carried out after crossing wild-type *D. subobscura *males with virgin females from the *ch-cu *marker strain. A total of 106 genes out of 306 differing in gene expression as concluded from the *a priori *contrasts were used as probes, and most of them (83%) yielded hybridization signals [see Additional file 3]. In all cases the negative results were due to failures in the amplifications. In no instance an exchange of genes among the different chromosomal elements of *D. melanogaster *and *D. subobscura *has been detected, which agrees with the well supported evidence for the established chromosomal homologies previously proposed for each Muller/Sturtevant/Novitski element [59]. The order of genes within each chromosomal element, however, is known to have widely changed among different species via the fixation of paracentric inversions (e.g.; [60-62]).

From the chromosomal homologies between *D. melanogaster *and *D. subobscura *[59], and the distribution of the ~120-megabase euchromatic portion of the *D. melanogaster *genome on each chromosomal arm [63], we tested the null hypothesis that the location of genes differing in gene expression between warm- and cold-adapted populations on the different *D. subobscura *chromosomes was at random (Table 2). The *G*-test for goodness of fit [64] detected a marginally nonsignificant random distribution (*p *= 0.059. If candidate genes *Hsp68 *and *Fst *on chromosome O, *Hsp60 *on chromosome U, and *Treh *on chromosome E are included, then *p *= 0.076), with chromosome J apparently being overrepresented and chromosome O underrepresented. It seems interesting to contrast these results with the previously reported chromosomal inversion shifts in the thermal populations [16]. Inversions on chromosomes J, E and, to a lesser extent, chromosomes A and O showed clear shifts in frequency according to the thermal regime, whereas those on chromosome U showed no trend whatsoever. It is not at all evident how the distribution of the differentially expressed genes on chromosomes matches with these patterns as, for example, chromosome E and U are well represented in Table 2 but their behaviour after two years of thermal evolution was completely different.

**Table 2:** Chromosomal distribution of genes differing in gene expression

Muller's chromosomal element	*D. melanogaster*	*D. subobscura*	Size of euchromatic portion (Mb; [63])	Genes differing in gene expression	
				Observed	Expected
				*f*	f^ MathType@MTEF@5@5@+=feaafiart1ev1aaatCvAUfKttLearuWrP9MDH5MBPbIqV92AaeXatLxBI9gBaebbnrfifHhDYfgasaacH8akY=wiFfYdH8Gipec8Eeeu0xXdbba9frFj0=OqFfea0dXdd9vqai=hGuQ8kuc9pgc9s8qqaq=dirpe0xb9q8qiLsFr0=vr0=vr0dc8meaabaqaciaacaGaaeqabaqabeGadaaakeaacuWGMbGzgaqcaaaa@2E11@
A	X	A	21.8	45	52.576
B	2L	U	23.0	63	55.471
C	2R	E	21.8	58	52.576
D	3L	J	24.4	70	58.847
E	3R	O	28.0	51	67.529
Sum			119	287	287

The *G*-test for goodness of fit [64] is G(4)=2∑i=15filog⁡e(fif^i)=9.08
 MathType@MTEF@5@5@+=feaafiart1ev1aaatCvAUfKttLearuWrP9MDH5MBPbIqV92AaeXatLxBI9gBaebbnrfifHhDYfgasaacH8akY=wiFfYdH8Gipec8Eeeu0xXdbba9frFj0=OqFfea0dXdd9vqai=hGuQ8kuc9pgc9s8qqaq=dirpe0xb9q8qiLsFr0=vr0=vr0dc8meaabaqaciaacaGaaeqabaqabeGadaaakeaacqWGhbWrdaWgaaWcbaGaeiikaGIaeGinaqJaeiykaKcabeaakiabg2da9iabikdaYmaaqahabaGaemOzay2aaSbaaSqaaiabdMgaPbqabaaabaGaemyAaKMaeyypa0JaeGymaedabaGaeGynaudaniabggHiLdGccyGGSbaBcqGGVbWBcqGGNbWzdaWgaaWcbaGaemyzaugabeaakmaabmaabaWaaSaaaeaacqWGMbGzdaWgaaWcbaGaemyAaKgabeaaaOqaaiqbdAgaMzaajaWaaSbaaSqaaiabdMgaPbqabaaaaaGccaGLOaGaayzkaaGaeyypa0JaeGyoaKJaeiOla4IaeGimaaJaeGioaGdaaa@4DF1@; *p *= 0.059.

**Figure 5:**
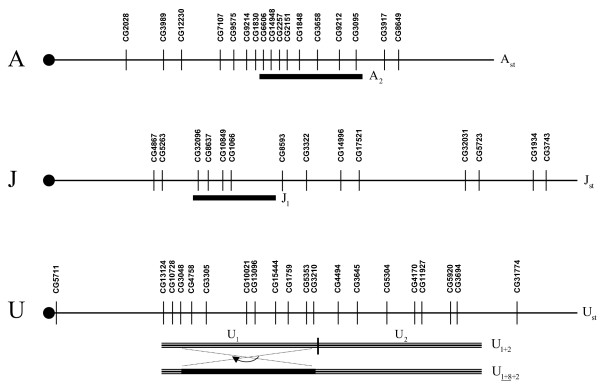
**Physical mapping of differentially expressed genes on chromosomes A, J, U of *Drosophila subobscura***. Schematic representation of the location of 17 differentially expressed genes mapped along chromosome A (the sex chromosome), 14 mapped along chromosome J (homologous to arm 3L in *D. melanogaster*), and 20 mapped along chromosome U (homologous to arm 2L in *D. melanogaster*). The centromere is placed on the left (black circle) and the telomere on the right. The linear order of genes is that in the standard gene arrangements, and the chromosomal regions covered by inversions segregating in the thermal stocks (labelled on the right-hand side next to the segments; overlapping inversions underlined) are indicated. (For further details on the formation of the gene arrangements by overlapping inversions, see [84].)

**Figure 6:**
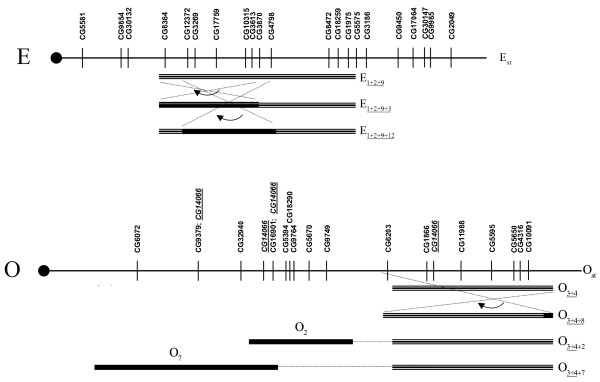
**Physical mapping of differentially expressed genes on chromosomes E and O of *Drosophila subobscura***. Schematic representation of the location of 20 differentially expressed genes mapped along chromosome E (homologous to arm 2R in *D. melanogaster*), and 16 mapped along chromosome O (homologous to arm 3R in *D. melanogaster*). The centromere is placed on the left (black circle) and the telomere on the right. The linear order of genes is that in the standard gene arrangements, and the chromosomal regions covered by inversions segregating in the thermal stocks are indicated (labelled on the right-hand side next to the segments; overlapping inversions underlined). Inversions O_5 _and O_7 _were also sporadically found but are ignored here: the first is associated to a lethal gene and the second is probably the result of a recombination event in the O_3+4+7_/O_st _heterokaryotype [16]).

Figs. 5, 6 show a schematic representation of the location of genes along the *D. subobscura *chromosomes, together with the chromosomal regions covered by the gene arrangements segregating in the thermal stocks. To test whether or not there is any clustering of genes within the inverted segments of the chromosomes we have relied on published tables where inversion lengths of *D. subobscura *are given as percentages of the total length of all chromosomes (except for the dot chromosome; [65]). Since overlapping inversions in chromosomes U, E and O introduce potential confusion, we have only considered the following heterokaryotypes in the analysis: A_st_/A_2_, J_st_/J_1_, U_st_/U_1+2_, U_1+2_/U_1+8+2 _(i.e., segment covered by inversion U_8_), E_st_/E_1+2+9_, E_1+2+9_/E_1+2+9+3_(segment covered by E_3_), E_1+2+9_/E_1+2+9+12_(segment covered by E_12_), O_st_/O_3+4_, O_3+4_/O_3+4+2 _(segment covered by O_2_), and O_3+4_/O_3+4+7 _(segment covered by O_7_). The *G*-test for goodness of fit detected a highly significant deviation from a random distribution (*G *_(9) _= 61.55; *p *< 0.001), with all but O_7 _chromosome segments covered by inversions having a higher than expected number of genes.

We further analyzed the correlated expression levels in cold- (P13) and warm-adapted (P22) populations and compared the correlation matrices of gene expression levels between P13 and P22 populations with a Mantel test [64] by randomly permuting 10,000 times the rows and columns of one of the matrices. For those physically mapped genes (Figs. 5, 6) the Mantel tests showed that correlated patterns of expression were similar for all chromosomes (ranging from Mantel's *r *= 0.418, *p *= 6.0 × 10^-4 ^for chromosome U to Mantel's *r *= 0.891, *p *= 2.0 × 10^-4 ^for chromosome J). The same conclusion was also obtained when simultaneously comparing the correlation patterns of the 306 differently expressed genes (Mantel's *r *= 0.783, *p *= 1.0 × 10^-4^).

To summarize, the physical mapping has shown that a larger than expected number of differentially expressed genes are located inside inverted chromosomal segments, and that the past thermal selection regime does not seem to have significantly changed the correlated patterns of gene expression. Since inversions in *D. subobscura *are know to influence temporal patterns of linkage disequilibrium between allozymes [66,67], disentangling the effects of inversions will be obviously required in any future approach if we want to identify the relevant candidate genes underlying thermal adaptation in *D. subobscura*.

## Conclusion

By looking at changes in the transcriptome this study has identified a large number of genes that appear to be involved in thermal adaptation in *Drosophila subobscura*, with a substantial fraction implicated in metabolism. Interestingly, expression levels of four previously reported candidate genes for thermotolerance in *Drosophila *(*Hsp26*, *Hsp68*, *Fst*, and *Treh*) were found to be correlated with past thermal selection regime. The highest experimental temperature used in our stocks (22°C) was not expected to be stressful. This was apparently confirmed by the lack of induction in the P22 populations of a heat shock response known to occur in *D. subobscura *[58]. The data also suggest an association with polymorphic inversions as some clustering of genes within inverted chromosomal sections was detected. This result is probably not surprising in view of the rapidly evolved latitudinal clines in inversion frequencies after the introduction of the species into the New World [12], as well as the quick response of inversion polymorphism to laboratory temperature [16]. The challenge now is to elucidate what associations are causal and what are due to correlated responses or hitchhiking arisen from linkage disequilibrium with the inversions.

## Methods

### Microarray experiment

#### a) Experimental populations and sampling protocol

All nine laboratory populations used here were initiated from an ancestral population of *Drosophila subobscura *derived from a large outbred stock collected in November 1999 at the estimated Chilean epicentre of the original New World invasion (Puerto Montt, Chile, 41° 28' S, 73° 00' W [68]). From that ancestral population three sets (P13, P18 and P22) of three replicate populations each (R1, R2 and R3) were set up in May 2001 and have since kept at three experimental temperatures on a discrete generation, controlled breeding under constant larval density (~5 larvae/mL of food) and constant 12:12 light:dark period: cold (13°C), optimum (18°C) and warm (22°C), respectively. The number of breeding adults per population is typically well over 1,500 flies. Complete details of the derivation and maintenance of these populations have been previously described [15,16].

Because we wanted to compare cDNA microarray gene expression patterns for all populations in a nested ANOVA design (see below), the use of a common reference mRNA to be competitively hybridized to treatment mRNAs coming from the different populations seemed to be more appropriate for statistical analysis than a design involving the correct pairing of all nine samples [69]. Therefore, the three 'optimum' P18 populations were sampled in April 2004 (two generations prior the experimental flies from the same populations were obtained) by placing a large number of eggs (± 4 h) in 120-mL plastic chambers (~100 eggs per chamber) with spoons containing 30 mL of David's killed-yeast *Drosophila *medium [70] stained with 0.05% bromophenol blue. This dye has no effect on larval growth and allows for accurate staging of third instar larvae just prior to pupation at their maximum size [71]. Larvae with clean guts that stopped feeding and started to wander on the wall of the plastic chambers were gently removed with a spatula, cleaned several times with distilled water, placed in bunches with 25 larvae each inside microcentrifuge tubes containing 500 *μ*L of TRI Reagent^® ^(Molecular Research Center, Inc.), and immediately homogenized and stored at -80°C. These larvae (hereafter referred to as C18) provided the mRNA used as reference.

Subsequently, samples from all nine populations were obtained in May-June 2004 (25 generations at 13°C, 35 at 18°C, and 46 at 22°C) by placing eggs into twelve 130-mL bottles (~200–250 eggs per bottle). These bottles were cultured at 18°C and emerging adults were dumped into Plexiglas cages for egg collections. Eggs for the experiment were collected over a six days period by placing Petri dishes containing non-nutritive agar with a generous smear of live yeast in the cages. As before, ~100 eggs (± 4 h) were placed at 18°C in 120-mL plastic chambers with stained *Drosophila *medium to sample the treatment third instar larvae for further mRNA extraction.

#### b) RNA extraction and gene expression analysis

Total RNA was extracted from the frozen homogenized larvae by using TRI Reagent^® ^(MRC, Inc.), and mRNA was extracted by using Promega PolyATtract^® ^isolation system following the manufacturer's specifications. Three mRNA extractions were performed from 9,000 C18 reference larvae (i.e., 3,000 larvae from each replicated population) to obtain a single reference pooled mRNA, and four independent extractions from 250 larvae each were made for each treatment population. This procedure ensured true replication in the experiment; namely, the reference mRNA was always hybridized with treatment mRNAs coming from independent larvae and extractions.

Relative mRNA levels were determined by parallel two-colour hybridization to cDNA microarrays from the Canadian *Drosophila *Microarray Centre (CDMC D12Kv1; [72]). The arrayed elements in these slides represent approximately 10,500 *D. melanogaster *genes to which seven *D. subobscura *genes were added as positive controls. As previously discussed, we were confident that the use of *D. melanogaster *arrays in heterologous hybridization with *D. subobscura *could offer a reasonable warranty to obtaining reliable data. However, the divergence between both species (~25 Mya; [29]) may have been the reason for the relatively high number of genes that failed to hybridize (Fig. 1), even though other factors like lack of gene expression in third instar larvae may have also been important. 1 μg of poly(A)^+^-enriched RNA was labelled using the SuperScript Indirect cDNA labelling system (Invitrogen Corporation, California, USA). mRNA concentrations and cDNA synthesis were checked with Agilent 2100 bioanalyzer (Agilent Technologies, California, USA). Equal amounts of labelled cDNA were combined with 10 μg of yeast tRNA, speed vac dried and re-suspended in 230 μl Dig Easy Hyb solution (Boehringer-Roche). The solution was incubated at 65°C for 10 min to denature the probes.

Hybridizations and washes were performed using the automatic system Lucidea SlidePro (Amersham, UK). The hybridization was allowed to proceed for 15 h at 25°C, and the slides were sequentially washed three times at 50°C for 10 min with medium stringency buffer (1 × SCC, 0.1% SDS), twice at room temperature for 1 min with high stringency buffer (1 × SCC), post wash buffer (0.1 × SCC) and air dried. Then each slide was scanned using an Axon GenePix 4000B microarray scanner (Axon Instruments, Union City, California, USA). Data were extracted from the scanned images using GenePix^® ^Pro (Axon Instruments) microarray image analysis. Labelling, hybridization and scanning were carried out at the Plataforma de Transcriptòmica from the Parc Cientific de Barcelona and Universitat de Barcelona (PCB-UB; [73]).

The raw data were adjusted using lowess normalization software (TIGR MIDAS ver. 2.19; [74]) with a tri-cube weight function and 0.33 smooth parameter applied to the C18 reference mRNA dye-labelled green (Cy3) or red (Cy5). For each experimental population four microarrays were independently hybridized and scanned, adding to 36 arrays in total. There were 14,440 duplicated spots on each array, and only the spots that passed a quality control of image analysis (i.e., array elements with intensities significantly different from background) were used in the differential expression analysis. The gene spots were further filtered by excluding those with less than 57 out of 72 (i.e., <79%) valid expression observations (Fig. 1), leaving 4,651 probes for differential gene expression analysis. The data acquired from these procedures were relative measures of gene expression independent of larval size differences among the thermal stocks.

#### c) Experimental design and data analysis

The unit of analysis here is the population, and the three replicated populations (R1, R2 and R3) of each thermal selection stock were treated as a random factor nested within experimental temperature (13, 18 and 22°C), which was a fixed effect [64]. Given any treatment population *pop *= 13R1, 13R2,⋯, 22R3, and any probe *g *= 1, ⋯, *G *for which valid expression levels were obtained, we use notation Zcont(G)g
 MathType@MTEF@5@5@+=feaafiart1ev1aaatCvAUfKttLearuWrP9MDH5MBPbIqV92AaeXatLxBI9gBaebbnrfifHhDYfgasaacH8akY=wiFfYdH8Gipec8Eeeu0xXdbba9frFj0=OqFfea0dXdd9vqai=hGuQ8kuc9pgc9s8qqaq=dirpe0xb9q8qiLsFr0=vr0=vr0dc8meaabaqaciaacaGaaeqabaqabeGadaaakeaacqWGAbGwdaqhaaWcbaGaem4yamMaem4Ba8MaemOBa4MaemiDaqNaeiikaGccbaGae83raCKaeiykaKcabaGaem4zaCgaaaaa@37C7@, Zcont(R)g
 MathType@MTEF@5@5@+=feaafiart1ev1aaatCvAUfKttLearuWrP9MDH5MBPbIqV92AaeXatLxBI9gBaebbnrfifHhDYfgasaacH8akY=wiFfYdH8Gipec8Eeeu0xXdbba9frFj0=OqFfea0dXdd9vqai=hGuQ8kuc9pgc9s8qqaq=dirpe0xb9q8qiLsFr0=vr0=vr0dc8meaabaqaciaacaGaaeqabaqabeGadaaakeaacqWGAbGwdaqhaaWcbaGaem4yamMaem4Ba8MaemOBa4MaemiDaqNaeiikaGccbaGae8NuaiLaeiykaKcabaGaem4zaCgaaaaa@37DD@ to denote normalized and background adjusted gene expression from the reference C18 mRNA sample that was dyed green (G) or red (R); and Zpop(G)g
 MathType@MTEF@5@5@+=feaafiart1ev1aaatCvAUfKttLearuWrP9MDH5MBPbIqV92AaeXatLxBI9gBaebbnrfifHhDYfgasaacH8akY=wiFfYdH8Gipec8Eeeu0xXdbba9frFj0=OqFfea0dXdd9vqai=hGuQ8kuc9pgc9s8qqaq=dirpe0xb9q8qiLsFr0=vr0=vr0dc8meaabaqaciaacaGaaeqabaqabeGadaaakeaacqWGAbGwdaqhaaWcbaGaemiCaaNaem4Ba8MaemiCaaNaeiikaGccbaGae83raCKaeiykaKcabaGaem4zaCgaaaaa@3674@, Zpop(R)g
 MathType@MTEF@5@5@+=feaafiart1ev1aaatCvAUfKttLearuWrP9MDH5MBPbIqV92AaeXatLxBI9gBaebbnrfifHhDYfgasaacH8akY=wiFfYdH8Gipec8Eeeu0xXdbba9frFj0=OqFfea0dXdd9vqai=hGuQ8kuc9pgc9s8qqaq=dirpe0xb9q8qiLsFr0=vr0=vr0dc8meaabaqaciaacaGaaeqabaqabeGadaaakeaacqWGAbGwdaqhaaWcbaGaemiCaaNaem4Ba8MaemiCaaNaeiikaGccbaGae8NuaiLaeiykaKcabaGaem4zaCgaaaaa@368A@ for gene expression intensities obtained from the treatment mRNAs dyed green or red. For each treatment population Zpop(G)g
 MathType@MTEF@5@5@+=feaafiart1ev1aaatCvAUfKttLearuWrP9MDH5MBPbIqV92AaeXatLxBI9gBaebbnrfifHhDYfgasaacH8akY=wiFfYdH8Gipec8Eeeu0xXdbba9frFj0=OqFfea0dXdd9vqai=hGuQ8kuc9pgc9s8qqaq=dirpe0xb9q8qiLsFr0=vr0=vr0dc8meaabaqaciaacaGaaeqabaqabeGadaaakeaacqWGAbGwdaqhaaWcbaGaemiCaaNaem4Ba8MaemiCaaNaeiikaGccbaGae83raCKaeiykaKcabaGaem4zaCgaaaaa@3674@/Zcont(R)g
 MathType@MTEF@5@5@+=feaafiart1ev1aaatCvAUfKttLearuWrP9MDH5MBPbIqV92AaeXatLxBI9gBaebbnrfifHhDYfgasaacH8akY=wiFfYdH8Gipec8Eeeu0xXdbba9frFj0=OqFfea0dXdd9vqai=hGuQ8kuc9pgc9s8qqaq=dirpe0xb9q8qiLsFr0=vr0=vr0dc8meaabaqaciaacaGaaeqabaqabeGadaaakeaacqWGAbGwdaqhaaWcbaGaem4yamMaem4Ba8MaemOBa4MaemiDaqNaeiikaGccbaGae8NuaiLaeiykaKcabaGaem4zaCgaaaaa@37DD@, Zpop(R)g
 MathType@MTEF@5@5@+=feaafiart1ev1aaatCvAUfKttLearuWrP9MDH5MBPbIqV92AaeXatLxBI9gBaebbnrfifHhDYfgasaacH8akY=wiFfYdH8Gipec8Eeeu0xXdbba9frFj0=OqFfea0dXdd9vqai=hGuQ8kuc9pgc9s8qqaq=dirpe0xb9q8qiLsFr0=vr0=vr0dc8meaabaqaciaacaGaaeqabaqabeGadaaakeaacqWGAbGwdaqhaaWcbaGaemiCaaNaem4Ba8MaemiCaaNaeiikaGccbaGae8NuaiLaeiykaKcabaGaem4zaCgaaaaa@368A@/Zcont(G)g
 MathType@MTEF@5@5@+=feaafiart1ev1aaatCvAUfKttLearuWrP9MDH5MBPbIqV92AaeXatLxBI9gBaebbnrfifHhDYfgasaacH8akY=wiFfYdH8Gipec8Eeeu0xXdbba9frFj0=OqFfea0dXdd9vqai=hGuQ8kuc9pgc9s8qqaq=dirpe0xb9q8qiLsFr0=vr0=vr0dc8meaabaqaciaacaGaaeqabaqabeGadaaakeaacqWGAbGwdaqhaaWcbaGaem4yamMaem4Ba8MaemOBa4MaemiDaqNaeiikaGccbaGae83raCKaeiykaKcabaGaem4zaCgaaaaa@37C7@ are the relative intensity ratios measured from the corresponding slides. The fully balanced dye-reversal experimental design can be written as the linear model:

yijklmg=μg+Tig+ℛj(i)g+Dkg+TDikg+Al(ijk)g+εijklmg,     (1)
 MathType@MTEF@5@5@+=feaafiart1ev1aaatCvAUfKttLearuWrP9MDH5MBPbIqV92AaeXatLxBI9gBaebbnrfifHhDYfgasaacH8akY=wiFfYdH8Gipec8Eeeu0xXdbba9frFj0=OqFfea0dXdd9vqai=hGuQ8kuc9pgc9s8qqaq=dirpe0xb9q8qiLsFr0=vr0=vr0dc8meaabaqaciaacaGaaeqabaqabeGadaaakeaacqWG5bqEdaqhaaWcbaGaemyAaKMaemOAaOMaem4AaSMaemiBaWMaemyBa0gabaGaem4zaCgaaOGaeyypa0dcciGae8hVd02aaWbaaSqabeaacqWGNbWzaaGccqGHRaWkcqWGubavdaqhaaWcbaGaemyAaKgabaGaem4zaCgaaOGaey4kaSYenfgDOvwBHrxAJfwnHbqeg0uy0HwzTfgDPnwy1aaceaGae43gHi1aa0baaSqaaiabdQgaQjabcIcaOiabdMgaPjabcMcaPaqaaiabdEgaNbaakiabgUcaRiabdseaenaaDaaaleaacqWGRbWAaeaacqWGNbWzaaGccqGHRaWkcqWGubavcqWGebardaqhaaWcbaGaemyAaKMaem4AaSgabaGaem4zaCgaaOGaey4kaSIae4haXh0aa0baaSqaaiabdYgaSjabcIcaOiabdMgaPjabdQgaQjabdUgaRjabcMcaPaqaaiabdEgaNbaakiabgUcaRiab=v7aLnaaDaaaleaacqWGPbqAcqWGQbGAcqWGRbWAcqWGSbaBcqWGTbqBaeaacqWGNbWzaaGccqGGSaalcaWLjaGaaCzcamaabmaabaGaeGymaedacaGLOaGaayzkaaaaaa@78AC@

where for probe *g*, *μ*^*g *^is the overall grand mean of the log 2 relative intensity ratios; Tig
 MathType@MTEF@5@5@+=feaafiart1ev1aaatCvAUfKttLearuWrP9MDH5MBPbIqV92AaeXatLxBI9gBaebbnrfifHhDYfgasaacH8akY=wiFfYdH8Gipec8Eeeu0xXdbba9frFj0=OqFfea0dXdd9vqai=hGuQ8kuc9pgc9s8qqaq=dirpe0xb9q8qiLsFr0=vr0=vr0dc8meaabaqaciaacaGaaeqabaqabeGadaaakeaacqWGubavdaqhaaWcbaGaemyAaKgabaGaem4zaCgaaaaa@30BC@ is the fixed effect of the *i*th experimental treatment (P13, P18, P22); ℛj(i)g
 MathType@MTEF@5@5@+=feaafiart1ev1aaatCvAUfKttLearuWrP9MDH5MBPbIqV92AaeXatLxBI9gBaebbnrfifHhDYfgasaacH8akY=wiFfYdH8Gipec8Eeeu0xXdbba9frFj0=OqFfea0dXdd9vqai=hGuQ8kuc9pgc9s8qqaq=dirpe0xb9q8qiLsFr0=vr0=vr0dc8meaabaqaciaacaGaaeqabaqabeGadaaakeaat0uy0HwzTfgDPnwy1egaryqtHrhAL1wy0L2yHvdaiqaacqWFBeIudaqhaaWcbaGaemOAaOMaeiikaGIaemyAaKMaeiykaKcabaGaem4zaCgaaaaa@3D6A@ is the random effect of the *j*th replicate population (R1, R2, R3) within treatment *i*; Dkg
 MathType@MTEF@5@5@+=feaafiart1ev1aaatCvAUfKttLearuWrP9MDH5MBPbIqV92AaeXatLxBI9gBaebbnrfifHhDYfgasaacH8akY=wiFfYdH8Gipec8Eeeu0xXdbba9frFj0=OqFfea0dXdd9vqai=hGuQ8kuc9pgc9s8qqaq=dirpe0xb9q8qiLsFr0=vr0=vr0dc8meaabaqaciaacaGaaeqabaqabeGadaaakeaacqWGebardaqhaaWcbaGaem4AaSgabaGaem4zaCgaaaaa@30A0@ is the fixed effect of dye *k *(Cy3, Cy5); TDikg
 MathType@MTEF@5@5@+=feaafiart1ev1aaatCvAUfKttLearuWrP9MDH5MBPbIqV92AaeXatLxBI9gBaebbnrfifHhDYfgasaacH8akY=wiFfYdH8Gipec8Eeeu0xXdbba9frFj0=OqFfea0dXdd9vqai=hGuQ8kuc9pgc9s8qqaq=dirpe0xb9q8qiLsFr0=vr0=vr0dc8meaabaqaciaacaGaaeqabaqabeGadaaakeaacqWGubavcqWGebardaqhaaWcbaGaemyAaKMaem4AaSgabaGaem4zaCgaaaaa@332C@ is the interaction term; Al(ijk)g
 MathType@MTEF@5@5@+=feaafiart1ev1aaatCvAUfKttLearuWrP9MDH5MBPbIqV92AaeXatLxBI9gBaebbnrfifHhDYfgasaacH8akY=wiFfYdH8Gipec8Eeeu0xXdbba9frFj0=OqFfea0dXdd9vqai=hGuQ8kuc9pgc9s8qqaq=dirpe0xb9q8qiLsFr0=vr0=vr0dc8meaabaqaciaacaGaaeqabaqabeGadaaakeaat0uy0HwzTfgDPnwy1egaryqtHrhAL1wy0L2yHvdaiqaacqWFaeFqdaqhaaWcbaGaemiBaWMaeiikaGIaemyAaKMaemOAaOMaem4AaSMaeiykaKcabaGaem4zaCgaaaaa@40CE@ is the random effect of slide *l *= 1, 2 within treatment *i*, replicate *j *and dye *k*; and εijklmg
 MathType@MTEF@5@5@+=feaafiart1ev1aaatCvAUfKttLearuWrP9MDH5MBPbIqV92AaeXatLxBI9gBaebbnrfifHhDYfgasaacH8akY=wiFfYdH8Gipec8Eeeu0xXdbba9frFj0=OqFfea0dXdd9vqai=hGuQ8kuc9pgc9s8qqaq=dirpe0xb9q8qiLsFr0=vr0=vr0dc8meaabaqaciaacaGaaeqabaqabeGadaaakeaaiiGacqWF1oqzdaqhaaWcbaGaemyAaKMaemOAaOMaem4AaSMaemiBaWMaemyBa0gabaGaem4zaCgaaaaa@36B9@ is the residual error associated with the corresponding log 2 relative intensity ratio of the *ijklm*th spot. This linear model easily allows partitioning all sources of experimental variation: biological (temperature and replicated population effects) and technical (dye and slide effects).

Notice that for the treatment effect we are interested in (i.e., the Tig
 MathType@MTEF@5@5@+=feaafiart1ev1aaatCvAUfKttLearuWrP9MDH5MBPbIqV92AaeXatLxBI9gBaebbnrfifHhDYfgasaacH8akY=wiFfYdH8Gipec8Eeeu0xXdbba9frFj0=OqFfea0dXdd9vqai=hGuQ8kuc9pgc9s8qqaq=dirpe0xb9q8qiLsFr0=vr0=vr0dc8meaabaqaciaacaGaaeqabaqabeGadaaakeaacqWGubavdaqhaaWcbaGaemyAaKgabaGaem4zaCgaaaaa@30BC@ component due to thermal adaptation) the linear model (1) can be conveniently reduced to the following two-level nested ANOVA model:

yijkg=μg+Tig+ℛj(i)g+eijkg,     (2)
 MathType@MTEF@5@5@+=feaafiart1ev1aaatCvAUfKttLearuWrP9MDH5MBPbIqV92AaeXatLxBI9gBaebbnrfifHhDYfgasaacH8akY=wiFfYdH8Gipec8Eeeu0xXdbba9frFj0=OqFfea0dXdd9vqai=hGuQ8kuc9pgc9s8qqaq=dirpe0xb9q8qiLsFr0=vr0=vr0dc8meaabaqaciaacaGaaeqabaqabeGadaaakeaacqWG5bqEdaqhaaWcbaGaemyAaKMaemOAaOMaem4AaSgabaGaem4zaCgaaOGaeyypa0dcciGae8hVd02aaWbaaSqabeaacqWGNbWzaaGccqGHRaWkcqWGubavdaqhaaWcbaGaemyAaKgabaGaem4zaCgaaOGaey4kaSYenfgDOvwBHrxAJfwnHbqeg0uy0HwzTfgDPnwy1aaceaGae43gHi1aa0baaSqaaiabdQgaQjabcIcaOiabdMgaPjabcMcaPaqaaiabdEgaNbaakiabgUcaRiabdwgaLnaaDaaaleaacqWGPbqAcqWGQbGAcqWGRbWAaeaacqWGNbWzaaGccqGGSaalcaWLjaGaaCzcamaabmaabaGaeGOmaidacaGLOaGaayzkaaaaaa@5B3B@

where the sum of squares for the error term eijkg
 MathType@MTEF@5@5@+=feaafiart1ev1aaatCvAUfKttLearuWrP9MDH5MBPbIqV92AaeXatLxBI9gBaebbnrfifHhDYfgasaacH8akY=wiFfYdH8Gipec8Eeeu0xXdbba9frFj0=OqFfea0dXdd9vqai=hGuQ8kuc9pgc9s8qqaq=dirpe0xb9q8qiLsFr0=vr0=vr0dc8meaabaqaciaacaGaaeqabaqabeGadaaakeaacqWGLbqzdaqhaaWcbaGaemyAaKMaemOAaOMaem4AaSgabaGaem4zaCgaaaaa@339A@ is simply the sum of the sum of squares for the remainder terms in (1). The usefulness of this model reduction is obvious to efficiently perform randomization tests to test the null hypothesis about treatment effects in a randomized (i.e., random assignment) experiment [75]. Permutation tests are far less sensitive to the presence of outliers and are particularly necessary with unequal sample sizes; i.e., when some data points are missing as is usually the case with microarray experiments. The null hypothesis of no treatment or evolutionary thermal regime effect was tested here after performing random permutations among replicate and selection temperature for the among selection temperature *F*-statistics. Each test used 10,000 random permutations of the log 2 relative intensity ratios (recall that when *N *= 72 there are 72!(24!24!24!)≈2.56×1032
 MathType@MTEF@5@5@+=feaafiart1ev1aaatCvAUfKttLearuWrP9MDH5MBPbIqV92AaeXatLxBI9gBaebbnrfifHhDYfgasaacH8akY=wiFfYdH8Gipec8Eeeu0xXdbba9frFj0=OqFfea0dXdd9vqai=hGuQ8kuc9pgc9s8qqaq=dirpe0xb9q8qiLsFr0=vr0=vr0dc8meaabaqaciaacaGaaeqabaqabeGadaaakeaadaWccaqaaiabiEda3iabikdaYiabcgcaHaqaaiabcIcaOiabikdaYiabisda0iabcgcaHiabikdaYiabisda0iabcgcaHiabikdaYiabisda0iabcgcaHiabcMcaPaaacqGHijYUcqaIYaGmcqGGUaGlcqaI1aqncqaI2aGncqGHxdaTcqaIXaqmcqaIWaamdaahaaWcbeqaaiabiodaZiabikdaYaaaaaa@44BF@ possible assignments of observations).

A planned comparison between the two treatment means from the stocks at the two extreme thermal regimes (i.e., P13 *vs*. P22) that had already diverged for 71 generations was also performed for each probe *g*. The permutation tests were performed following [76]; namely, we first calculated the Fg1
 MathType@MTEF@5@5@+=feaafiart1ev1aaatCvAUfKttLearuWrP9MDH5MBPbIqV92AaeXatLxBI9gBaebbnrfifHhDYfgasaacH8akY=wiFfYdH8Gipec8Eeeu0xXdbba9frFj0=OqFfea0dXdd9vqai=hGuQ8kuc9pgc9s8qqaq=dirpe0xb9q8qiLsFr0=vr0=vr0dc8meaabaqaciaacaGaaeqabaqabeGadaaakeaacqWGgbGrdaqhaaWcbaGaem4zaCgabaGaeGymaedaaaaa@3035@ statistic for the observed data and next the residuals of the log 2 relative intensity ratios from the populations at P13 and P22 were randomly allocated to both treatment temperatures. From *B *= 10,000 random permutations we got a set of null statistics Fg0b
 MathType@MTEF@5@5@+=feaafiart1ev1aaatCvAUfKttLearuWrP9MDH5MBPbIqV92AaeXatLxBI9gBaebbnrfifHhDYfgasaacH8akY=wiFfYdH8Gipec8Eeeu0xXdbba9frFj0=OqFfea0dXdd9vqai=hGuQ8kuc9pgc9s8qqaq=dirpe0xb9q8qiLsFr0=vr0=vr0dc8meaabaqaciaacaGaaeqabaqabeGadaaakeaacqWGgbGrdaqhaaWcbaGaem4zaCgabaGaeGimaaJaemOyaigaaaaa@3180@, *b *= 1,2, ..., *B*; and the *p*-value was computed as:

pg=∑b=1B#{[Fg1,Fg0b]≥Fg1}B+1.     (3)
 MathType@MTEF@5@5@+=feaafiart1ev1aaatCvAUfKttLearuWrP9MDH5MBPbIqV92AaeXatLxBI9gBaebbnrfifHhDYfgasaacH8akY=wiFfYdH8Gipec8Eeeu0xXdbba9frFj0=OqFfea0dXdd9vqai=hGuQ8kuc9pgc9s8qqaq=dirpe0xb9q8qiLsFr0=vr0=vr0dc8meaabaqaciaacaGaaeqabaqabeGadaaakeaacqWGWbaCdaWgaaWcbaGaem4zaCgabeaakiabg2da9maaqahabaWaaSaaaeaacqGGJaWidaGadeqaamaadmaabaGaemOray0aa0baaSqaaiabdEgaNbqaaiabigdaXaaakiabcYcaSiabdAeagnaaDaaaleaacqWGNbWzaeaacqaIWaamcqWGIbGyaaaakiaawUfacaGLDbaacqGHLjYScqWGgbGrdaqhaaWcbaGaem4zaCgabaGaeGymaedaaaGccaGL7bGaayzFaaaabaGaemOqaiKaey4kaSIaeGymaedaaaWcbaGaemOyaiMaeyypa0JaeGymaedabaGaemOqaieaniabggHiLdGccqGGUaGlcaWLjaGaaCzcamaabmaabaGaeG4mamdacaGLOaGaayzkaaaaaa@5274@

Given the high-dimensionality of the data set the *p*-values were adjusted based on the concept of false discovery rate (FDR; [34]). If no probe *g *is differentially expressed the *p*-values will follow a *U *(0,1), where *U *stands for 'uniform distribution'. The so-called Mixture Distribution Partitioning (MDP) methodology assumes that the distribution of *p*-values consists of a set of null *p*_0 _and alternative *p*_1 _components. This partition forms the basis for estimating various quantities as for example the *q*-values, which were obtained here with the QVALUE software [35]. The problem now is to select a threshold of significance to identify a set of genes likely to be differentially expressed. As an unsupervised criterion we used a *q*-value cut-off ≤ 0.05 for the P13 *vs*. P22 planned comparisons, meaning that the maximum expected proportion of false positives incurred when calling a particular gene 'differentially expressed' is 5%.

#### d) Computer software for statistical analysis

The computer programs used for statistical data analyses were MATLAB algebra program environment (ver. 7.0.4 [77]) together with the collection of tools supplied by the Statistics Toolbox (ver. 5.0.2 [78]). The statistical software packages STATISTICA version 6 [79] and SPSS version 13 [80] were also used.

### Mapping of candidate genes

The flies used for physical mapping of candidate genes were collected from a natural population in Bordils (70 Km North-east of Barcelona, Spain; 42° 3' N, 2° 54' E). About 150 males were individually crossed to three or four virgin females from the *ch-cu *marker strain to help in the identification of polymorphic inversions (the genetic background of this strain is highly homogeneous and fixed for the standard arrangements in all major acrocentric chromosomes but chromosome O, where it is fixed for arrangement O_3+4_.

DNA isolation, DNA amplification, polytene chromosome preparation and *in situ *hybridization were carried out using standard techniques [81]. The karyotype of *D. subobscura *consists of five acrocentric chromosomes and a dot chromosome. Following [82] the large chromosomes in this species are traditionally named as A (= X, the sex chromosome), J (= chromosomal element D of Mueller/Sturtevant/Novitski and homologous to arm 3L in *Drosophila melanogaster *[59]), U (= chromosomal element B and homologous to arm 2L), E (= chromosomal element C and homologous to arm 2R), and O (= chromosomal element E and homologous to arm 3R). The five major acrocentric chromosomes and the dot chromosome are divided into 100 sections (A: 1 – 16; J: 17 – 35; U: 36 – 53; E: 54 – 74; O: 75 – 99; Dot :100), and each section into 3–5 subsections (A, B, ...) [83].

## Authors' contributions

HL sampled the thermal populations, made the RNA extractions, participated in the design of the experiment, carried out statistical analysis, Gene Ontology searches, and drafted the manuscript. FG-F, BEC-S and VT designed primers, carried out *in situ *hybridizations on the polytene chromosomes of *D. subobscura*, and read all salivary gland squashes from the *in situ *hybridizations. SB and MC coordinated the microarray experiments for generating the original data. MS set up and maintained the thermal populations, designed the study, carried out statistical analyses and drafted the final version of the manuscript. All authors read and approved the final manuscript.

## Supplementary Material

Additional file 1 **Summary of microarray results from the 4,651 cDNA clones with valid expression observations**. The details for the different columns are explained within the file itself. Click here for file

Additional file 2 **Molecular function GO categories for the differentially expressed genes**. Analysis of functional categories defined by the Gene Ontology project [36] using the GOToolBox [37]. Click here for file

Additional file 3 **Physical map of differentially expressed genes**. Localization by *in situ *hybridization on the salivary gland chromosomes of *Drosophila subobscura *of 88 differentially expressed genes. Click here for file
