# Supplementary material for: Thermal evolution of gene expression profiles in Drosophila subobscura
Source: BMC Evol Biol. 2007 Mar 19;7:42. doi: 10.1186/1471-2148-7-42 (PMC1847442; doi:10.1186/1471-2148-7-42)
Supplement: Additional file 3 — Physical map of differentially expressed genes. Localization by in situ hybridization on the salivary gland chromosomes of Drosophila subobscura of 88 differentially expressed genes. [file 1471-2148-7-42-S3.pdf]

Additional file 3: Physical map of differentially expressed genes.

Localization by *in situ* hybridization on the salivary gland chromosomes of *Drosophila subobscura* of 88 differentially expressed genes. The five major acrocentric chromosomes and the dot chromosome in this species are divided into 100 sections ($\mathrm{A}:1-16;\ \mathrm{J}:17-35;\ \mathrm{U}:36-53;\ \mathrm{E}:54-74;\ \mathrm{O}:75-99;\ \mathrm{Dot}:100$), and each section into 3-5 subsections (A, B, ...). ('Size' refers to the length of the amplified fragment.)

Gene (Symbol)	Primers	Size (bp)	Hybridization signal
**Chromosome A**			
CG2028 (*Cklalpha*)	5′-ATGAGCATCCAGAGCGGCGA-3′<br>5′-CTTGCCGTTCTGCTTCTCCT-3′	981	A(3D)
CG3989 (*ade5*)	5′-CTTGGCAAGGTTCTGATCGA-3′<br>5′-GGATAGAGAACGGTGGCACA-3′	1717	A(5A)
CG12230 (*car*)	5′-CTCATCAGGCTGTCGAGGAA-3′<br>5′-CGATCAGTTGATACTGCTGG-3′	1219	A(5D)
CG7107 (*up*)	5′-GGCCGGTGTATTGTTCCTTC-3′<br>5′-GAAGGACAAGGACAAGAAGG-3′	2037	A(7B)
CG9575 (*Rab35*)	5′-GTGTCCTTCTGCTGCTCGTT-3′<br>5′-GCTTATAATCGGCGACAGCG-3′	1310	A(7D)
CG9214 (*Tob*)	5′-CGTTCCACTGCTTCTCCATA-3′<br>5′-CGCCGAATATGTTCACACGT-3′	2172	A(8B)
CG1830 (*PhKgamma*)	5′-TAATGGCCAAGGATGAGGAG-3′<br>5′-CGAACATGTTGCACTTGAGC-3′	1324	A(8C)
CG6606 (*l(1)G0003*)	5′-CACCAACAACTGCTTCGTCA-3′<br>5′-CTCATTCAGCCGCAGCTCTG-3′	1871	A(8D)
CG14948 (*dpr18*)	5′-TGGAGATGCGACTAGTTAGC-3′<br>5′-GATGCCGGTATCTACATGTG-3′	568	A(8E)
CG2257 (*Ubc-E2H*)	5′-CGCATGGACAATGACGTGAT-3′<br>5′-ACACAGGTGGCATAAGTTAG-3′	1984	A(9A)
CG2151 (*Trxr-1*)	5′-TAGTGGAGACCATAGACGCG-3′<br>5′-CCAAGAAGCTGATGCACCAG-3′	1226	A(9B)
CG1848 (*LIMK1*)	5′-AAGTTGCGCACGGAGTTCTG-3′<br>5′-TTCTCGCCTATGACCAGATC-3′	1742	A(10A)
CG3658 (*CDC45L*)	5′-CGCATCCTCATCATCGTCAA-3′<br>5′-AGCTGGACAACCGAAGGCTC-3′	1639	A(10D)
CG9212 (*Nipsnap*)	5′-GGCACAGTGTAGGCTACACA-3′<br>5′-GGCACAGTGTAGGCTACACA-3′	587	A(11C)
CG3095 (*hfw*)	5′-CTCCTGGTATTGCTGCAACA-3′<br>5′-GTCCAATCAGGCGGTGACTG-3′	1247	A(12A)
CG3917 (*Grip84*)	5′-ACCTACAGCCGACCATCTGG-3′<br>5′-CAGCATGCAGTTCTTGAGGC-3′	1280	A(13A)
CG8649 (*Fim*)	5′-ACTTACTCTTCTCCTCGCGC-3′<br>5′-CTATCAGGTGCGCGAGATGA-3′	1036	A(13E)

Additional file 3: (Continued.)

Gene (Symbol)	Primers	Size (bp)	Hybridization signal
**Chromosome J**			
CG4867 (*bc10*)	5′-TGCTGCCGGTGCTCCTCATA-3′<br>5′-TTGCATGTGCACACACGACG-3′	924	J(20E)
CG5263 (smg)	5′-CAACTGGCACAACAGACAAC-3′<br>5′-GCACAATGTGTGTTAGCTCC-3′	2135	J(21A)
CG32096 (*rols*)	5′-ACCTCGACAACCTCACAGAG-3′<br>5′-TCAAGTCCTCCAGCTACAAG-3′	2146	J(22C)
CG8637 (*trc*)	5′-GTGATGGTGTTAGAGAGACG-3′<br>5′-GATGTTCTTGTGGAGGCCGA-3′	1671	J(22E)
CG10849 (*Sc2*)	5′-TTGTAGTTGCGATGCTTGGC-3′<br>5′-GCAAGAGCCTGAAGGATACG-3′	1058	J(23B)
CG1066 (*Shab*)	5′-ACTTATGGTCGGCGAACGCG-3′<br>5′-CTCCTTGCGCATCTCCTCGT-3′	1407	J(23C)
CG8593 (*qm*)	5′-GACAATTCGATCCTTCGCAG-3′<br>5′-AAGAGGCTCCGCCATTCGCA-3′	1110	J(25D)
CG3322 (*LanB2*)	5′-CCTACCAGTACTACAGTGCC-3′<br>5′-ATGCTGCGGCTGTACTGGTT-3′	1816	J(26C)
CG14996 (*Chd64*)	5′-TCTACATGTGCCTGGTGTTG-3′<br>5′-TAGCAGAGCAGAGAGCCATG-3′	968	J(27E)
CG17521 (*Qm*)	5′-AGTAGCGAAGCCTTGGAAGC-3′<br>5′-CATAGCGCTCTCGGTCGTAC-3′	1074	J(28B)
CG32031 (*Argk*)	5′-CATGGTTGACGCTGCTGTTC-3′<br>5′-AAGGCGGACGAGCTGAAGAT-3′	1227	J(32A)
CG5723 (*Ten-m*)	5′-CGACTACGACCTGATGAAGC-3′<br>5′-TAGTTGTCCGGCGTGCTCAG-3′	1946	J(32C)
CG1934 (*ImpE2*)	5′-CTTGGTGATACTCTGCCTGG-3′<br>5′-TCGCTGGCATCGTCATTGAC-3′	1403	J(34D)
CG3743 (*MTF-1*)	5′-GAAGACGGATGCAACAAGGC-3′<br>5′-CATCCTCGATGTTCTGGTTC-3′	1496	J(35A)
**Chromosome U**			
CG5711 (*Arr1*)	5′-CTCACAGTGTGGTCGATGCC-3′<br>5′-ATGAGTACGTGCGCCAGAAC-3′	791	U(36A)
CG13124	5′-CCGTCTCCTTGCAGAGCTAA-3′<br>5′-GGAGGCCTTCAGCTCGATGA-3′	1322	U(40A)
CG10728	5′-ACTGCAGGTCTGGTCCACAT-3′<br>5′-TGTCCAATGGTAAGTAGTGT-3′	817	U(40B)
CG3048 (*Traf1*)	5′-GGCTGCAAGTGGTCTGATGA-3′<br>5′-TCCAGCACTGCAAGAACCTA-3′	1403	U(40C)

Additional file 3: (Continued.)

Gene (Symbol)	Primers	Size (bp)	Hybridization signal
CG4758 (*Trp1*)	5′-AGCCATCGAAGGACGAGAAG-3′<br>5′-TCAGATCAGAATTGCCTAGC-3′	2583	U(41B)
CG3305	5′-GATAGTGTGTGCGACAGCCA-3′<br>5′-CCACATCTGAGGTCTCCGAG-3′	454	U(41D)
CG10021 (*bowl*)	5′-CACTCGTCTCTGTGCTCGGT-3′<br>5′-ACAAGTGTCCTGTGTGCAGT-3′	1160	U(43A)
CG13096	5′-TCGTCTTCATCATCGTCATC-3′<br>5′-TGGCGGAGAATATTCTACTG-3′	621	U(43B)
CG15444 (*ine*)	5′-GCAGGAAGTTGATTAGCGCA-3′<br>5′-CTCGCTAGTGTGGTCGTCTC-3′	1524	U(44B)
CG1759 (*cad*)	5′-CACTACTACAATACACTGCC-3′<br>5′-TGGATATCGTCGATATTGTC-3′	2442	U(44D)
CG5353 (*Aats-thr*)	5′-AGGTGAACGGACAGGTCTGG-3′<br>5′-GCACATTGACGGTATTCGAG-3′	1761	U(45C)
CG3210 (*noodle*)	5′-TCCGACTTGTACAGATGTGT-3′<br>5′-AAGAGTTCGGTTATCGAGAG-3′	2289	U(45D)
CG4494 (*smt3*)	5′-GTCTGCTGCTGGTACACCTC-3′<br>5′-CGGTAAGAACGCAAGATTAA-3′	439	U(46C)
CG3645	5′-CTGGTCTGCCGATTAACAGT-3′<br>5′-GGATCTAGGCCTGGCTTGAG-3′	2065	U(47A)
CG5304 (*l(2)01810*)	5′-TTGAACGGCTGTACCTCTCC-3′<br>5′-AAGAAGAACGTCACGGATGA-3′	1435	U(48B)
CG4170 (*vig*)	5′-GTATACCAAGAACACATGTG-3′<br>5′-CGCTTCCTGTTCTGTCCATC-3′	1197	U(49B)
CG11927	5′-CCTTCACGAAGCTATTGCTG-3′<br>5′-AACAACAGCAGCGGACGTAA-3′	1329	U(49C)
CG5920 (*sop*)	5′-TGATAGACCTACATGTAAGC-3′<br>5′-CTTGGACAAGAAGTCAGAGT-3′	1164	U(50D)
CG3694 (*Ggamma30A*)	5′-TAGATCGATCTCATACCAAG-3′<br>5′-ACAGAATGCGATCGTTCATC-3′	718	U(50E)
CG31774 (*fred*)	5′-GATGGTCAGCAATGTCCGAT-3′<br>5′-TTGATGACAGCCTCCTTGAA-3′	1949	U(53A)
**Chromosome E**			
CG5581 (*Ote*)	5′-AATGGCCGACGTGGACGATT-3′<br>5′-TGGGCTTTATTGGAACACGG-3′	1424	E(55B)
CG9854 (*hrg*)	5′-CAATGGCAATTCTACCTCGG-3′<br>5′-GGTTGTCACGAACATGCCAC-3′	2247	E(57A)
CG30132 (*par-1*)	5′-CCAAGGAATGTTCCATCACG-3′<br>5′-GAGAGCGTAGAGAGGAAGCG-3′	3400	E(57B)

Additional file 3: (Continued.)

Gene (Symbol)	Primers	Size (bp)	Hybridization signal
CG8364 (*Rep3*)	5′-GATACACCTGGACTGCGAT-3′<br>5′-GCTATTGGTCTAGGAACAAT-3′	744	E(59A)
CG12372 (*spt4*)	5′-CAATTTGAGACTGACGGCTG-3′<br>5′-CCGAAGAGTGGGAGAAAGTG-3′	1997	E(60B)
CG3269 (*Rab2*)	5′-CCTTGCTGGTGTACGACATC-3′<br>5′-ATTGATCATTGAATCGCAGC-3′	1386	E(60C)
CG17759 (*Galpha49B*)	5′-AATCGAGAAGCAGTTGCGCC-3′<br>5′-TCGATTGCAGAATGGTGTCC-3′	3240	E(61D)
CG10315 (*elF2B-delta*)	5′-AGCCTGTTCATCAACGATCC-3′<br>5′-GGATCGCTCAGTTCGTTGTA-3′	856	E(63A)
CG3613 (*qkr58E-1*)	5′-CGCGCGACTACGATAGAGAC-3'<br>5′-AGCAGATGAATGTGATCCCG-3'	2067	E(63B)
CG3870 (*chrw*)	5′-CAACATCATCTTGGACGAGG-3′<br>5′-TGTTGGTGTTGGTGTAGGCC-3′	1610	E(63C)
CG4798 (*l(2)k01209*)	5′-CCTGTTGTCCATGGACTGTT-3′<br>5′-TAATCTCCGGATCTAGAGCG-3′	1439	E(64B)
CG8472 (*Cam*)	5′-AATTCCTGCTAGACGCCGCC-3′<br>5′-ATCATGTCCTGCAGCTCGGC-3′	2806	E(67A)
CG1975 (*Rep2*)	5′-AGAGTTCGCTGGACAACAAG-3′<br>5′-CAACACTACAAGTAACGGCA-3′	1832	E(68A)
CG5575 (*ken*)	5′-GCAATACAGCAAGCATGGCG-3′<br>5′-CGACTCGAAGCACATGGAGC-3′	1676	E(68C)
CG3186 (*eIF-5A)*	5′-GGCCATGCAAAATTGTCGAG-3′<br>5′-GCAATAACGCACTCCTCTCC-3′	689	E(69A)
CG9450 (*tud*)	5′-GACAACGACGACGACAAGAC-3′<br>5′-TGCCGCACAATTACAGAGTC-3′	2533	E(70D)
CG17064 (*gkap*)	5′-ATTGCGAGGAGGACATGTCG-3′<br>5′-GTGATCAACATGGCCAATGG-3′	2905	E(71C)
CG30147 (*Hil*)	5′-CTTGTCTTCTTACGGCGTGC-3′<br>5′-CGAGAGCATCTTCCTGGACG-3′	3083	E(72A)
CG9985 (*sktl*)	5′-GGTAACCTACAAGAAGATCC-3′<br>5′-CTTAGAATACGTAAACGCAC-3′	2539	E(72B)
CG2049 (*Pkn*)	5′-GGCGAATACATCAAGCATCC-3′<br>5′-ATCTGCAGCTGCTTCTCCAG-3′	2218	E(73B)
**Chromosome O**			
CG6072 (*sra*)	5′-GCCAGAGCTGCCAGTAGATC-3′<br>5′-CGGCCAAGTCCAATAACAAT-3′	726	O(79C)
CG9379 (*by*)	5′-CTGGTTATCCTTGGAGCTGT-3′<br>5′-AAGAATGCCTATGGCCTGGT-3′	590	O(82B)

Additional file 3: (Continued.)

Gene (Symbol)	Primers	Size (bp)	Hybridization signal
CG14066 (*larp*)	5′-CCGACTAATCGCATACCACG-3′<br>5′-ATCAATCGAGTGCGAATTGG-3′	2111	O(82B); (85B); (85E); (93C)
CG32940 (*Pif1*)	5′-AATGTATCACAAGGAGAACG-3′<br>5′-CTCCTGGTAGTACTGCAGAT-3′	2241	O(84A)
CG16901 (*sqd*)	5′-AACTAACCTTGTTCCTCTCC-3′<br>5′-TTACACACGCTTCGTCAGTT-3′	2892	O(85E)
CG5394 (*Aats-glupro*)	5′-GTTAGCAGCTGGCCGTACTC-3′<br>5′-TGTTGGTCATGTTCAAGCGA-3′	1692	O(86C)
CG18290 (*Act87E*)	5′-TTAGAAGCACTTGCGGTGGA-3′<br>5′-CCAGTCCAAGAGAGGTATCC-3′	954	O(86D)
CG9764 (*yrt*)	5′-CTGGACATCATCGAGAAGGA-3′<br>5′-ACATTGGCCAGCTTCACTTG-3′	2143	O(86E)
CG5670 (*Atpalpha*)	5′-TCATAAGATCTCTCCTGAGG-3′<br>5′-GCAATATCCTCAACGGTCTC-3′	1959	O(87C)
CG9749 (*Abi*)	5′-ACCTTCTCCTTGTGAATATG-3′<br>5′-GTGAGTAGTAATTAGGTTCG-3′	534	O(88B)
CG6203 (*Fmr1*)	5′-ACAGCCAAGTCGTTCTACCA-3′<br>5′-CCATTCACCAGACCTTCCTT-3′	2280	O(91B)
CG1866 (*Moca-cyp*)	5′-GCTCCTTCTCGATATCGTCC-3′<br>5′-GTAGTACAACACAGCCTGCG-3′	2483	O(93A)
CG11988 (*neur*)	5′-CATCATCCGCATCAGCAGTC-3′<br>5′-GAGTTGTGTTGCAGCATCTT-3′	788	O(94D)
CG5595 (*Sce*)	5′-AAGCCGCAGGAGATAATCAC-3′<br>5′-ACCTGATGCAACGTCTGGTT-3′	1192	O(96A)
CG5650 (*Pp1-87B*)	5′-TTCATGCTAGTTAGCTGTGA-3′<br>5′-GCTTCAAGATCTGGAACGAG-3′	1018	O(97A)
CG4316 (*Sb*)	5′-GTGGCTATCCAGTTCTCGTT-3′<br>5′-CCAGAACTTCAAGATCAGCC-3′	2288	O(97B)
CG10091 (*GstD9*)	5′-AATCCTCAGCACACGATTCC-3′<br>5′-TTCTTCGCATTCTCGTACCA-3′	430	O(97C)
